# Human exposure and stability of a novel *Citrus* flavanone-based nutraceutical: a multi-matrix analytical approach

**DOI:** 10.3389/fphar.2026.1810201

**Published:** 2026-05-20

**Authors:** Antonella Smeriglio, Eleonora Agostino, Souda Belaid, Mariarosaria Ingegneri, Paola Gualtieri, Matteo Salina, Sara Langone, Diego Bosco, Laura Di Renzo, Domenico Trombetta

**Affiliations:** 1 Department of Chemical, Biological, Pharmaceutical and Environmental Sciences, University of Messina, Messina, Italy; 2 Section of Food Science, Clinical Nutrition and Pharmaceutical Sciences, Department of Biomedicine and Prevention, University of Tor Vergata, Rome, Italy; 3 Consorzio Italbiotec, Milano, Italy; 4 Italbiotec S.r.l. Società Benefit, Milano, Italy

**Keywords:** *Citrus* flavanones, flavanone disposition, formulation stability, human exposure, intestinal metabolism, mass balance, multi-matrix analysis, nutraceuticals

## Abstract

**Introduction:**

Citrus flavanone glycosides are widely investigated for their potential health benefits, including antioxidant, anti-inflammatory, and cardiometabolic effects; however, human exposure and metabolic fate are often inferred from plasma measurements alone, despite limited systemic absorption and extensive intestinal processing.

**Methods:**

In this study, we performed an integrated, translational assessment of human exposure to a novel Citrus flavanone-based nutraceutical formulation (FlavonAid™), combining plasma, urinary, and fecal analyses with comprehensive analytical characterization and stability evaluation. Six healthy volunteers received a single oral dose (174 mg total flavanones), and flavanone-related compounds were quantified in plasma, urine, and feces using validated chromatographic methods.

**Results:**

Accelerated stability testing demonstrated minimal degradation (<3.5% over 6 months) and high reliability of the formulation. Plasma analysis revealed low and intermittent systemic exposure, with concentrations frequently close to analytical limits and no reproducible pharmacokinetic profiles. In contrast, urinary and fecal analyses showed substantial recovery of flavanone-related compounds within 24 h. Mass balance analysis indicated that approximately 39% of the administered dose was recovered in urine and feces during the first 24 h, mainly as hydrolyzed parent flavanone glycoside equivalents, while shared low-molecular-weight phenolic metabolites accounted for a major fraction of the excreted material.

**Discussion:**

Overall, these findings demonstrate that human exposure to Citrus flavanones is best described through a multi-matrix approach that captures coordinated intestinal metabolism, partial systemic handling, and prolonged elimination rather than relying solely on plasma data. This study establishes a robust methodological framework for evaluating flavanone-based nutraceuticals in humans and supports further investigation of formulations designed to achieve sustained intestinal exposure with limited systemic availability.

## Introduction

*Citrus* fruits represent one of the main dietary sources of flavanones, a class of flavonoids widely studied for their potential biological effects. The predominant *Citrus* flavanone glycosides, including hesperidin, narirutin and naringin, are widely consumed through fresh fruits, juices and derived products, resulting in a continuous and substantial human exposure (Manach et al., 2003; Brett et al., 2009).

Despite this widespread intake, the pharmacokinetic behaviour of *Citrus* flavanones in humans remains complex and not fully elucidated. Due to their glycosylated structure, flavanone glycosides are poorly absorbed in the small intestine and reach the colon largely intact, where they undergo extensive microbial metabolism (Manach et al., 2003; Brett et al., 2009). Consequently, systemic exposure to native flavanones is typically low, and parent compounds are rarely detected in plasma following oral intake of *Citrus* products (Manach et al., 2003).

Most of the available evidence on *Citrus* flavanones derives from *in vitro* and animal studies, often using doses and forms that do not reflect human dietary exposure. These studies have provided important mechanistic insights into antioxidant, anti-inflammatory and cardiometabolic effects; however, their translational relevance to humans remains limited.

In contrast, human studies addressing flavanone metabolic fate and systemic exposure remain scarce and often restricted to single biological matrices or short pharmacokinetic windows (Brett et al., 2009; Najmanová et al., 2020).

An additional level of complexity arises from the central role of the intestinal microbiota. In the colon, microbial enzymes hydrolyze flavanone glycosides, releasing aglycones that are further transformed into a wide range of low-molecular-weight phenolic metabolites (Mikropoulou et al., 2025). Following absorption, these compounds undergo extensive phase II metabolism, mainly glucuronidation and sulfation, in enterocytes and hepatocytes. Consequently, flavanone-derived compounds circulate mainly as conjugated metabolites, while free aglycones are rarely detected in plasma (Brett et al., 2009; Najmanová et al., 2020). Interindividual variability in gut microbiota composition further contributes to marked differences in metabolic profiles and exposure levels among subjects (Nishioka et al., 2021).

Within this framework, classical pharmacokinetic approaches based on plasma quantification of parent compounds appear inadequate to describe the true extent of human exposure to *Citrus* flavanones. Low or undetectable plasma concentrations do not necessarily indicate poor bioavailability or lack of biological relevance but rather reflect the dominance of metabolic and microbial transformations that are not accounted for by conventional pharmacokinetic metrics (Najmanová et al., 2020). Alternative matrices, such as urine and feces, can provide complementary and often more informative insights into flavanone metabolism and exposure, as they integrate absorption, biotransformation and excretion processes over time (Brett et al., 2009).

However, human studies adopting a multi-matrix approach, simultaneously investigating plasma, urine and feces, are still extremely limited. This represents a critical gap in the current literature, particularly considering that such an approach is essential to fully understand the metabolic fate of flavanones and the contribution of the intestinal microbiota to their systemic availability.

In this context, the present study aimed to investigate human exposure to *Citrus* flavanone glycosides, following administration of a novel nutraceutical formulation (FlavonAid™), by combining pharmaceutical characterization and stability assessment with a human intervention study.

By adopting an integrated multi-matrix analytical strategy, this study aims to overcome the limitations of classical pharmacokinetics and to provide a more realistic and comprehensive interpretation of flavanone exposure in humans, thereby brinding the gap between preclinical evidence and human data.

## Materials and methods

### Nutraceutical characterization, accelerated stability and shelf-life

#### Composition of the nutraceutical formulation

FlavonAid™ is a proprietary *Citrus* flavanone-based formulation for oral use. Each capsule contains pharmaceutical-grade hesperidin and standardized *Citrus* extracts providing flavanone glycosides, including eriocitrin, neoeriocitrin, naringin, and neohesperidin. During the human study, the formulation was administered at a dose of one capsule per day, corresponding to a total daily intake of 174 mg of *Citrus* flavanones. Detailed technological and manufacturing aspects are covered by intellectual property protection (European Patent Application No. EP24218895.1).

#### Analytical characterization of flavanone glycosides

The flavanone glycoside profile of the formulation was characterized prior to the human study to define its qualitative and quantitative composition. The analysis focused on the main *Citrus* flavanone glycosides, namely, hesperidin, eriocitrin, neoeriocitrin, naringin, and neohesperidin.

Chromatographic separation was achieved by high-performance liquid chromatography coupled with diode-array detection (HPLC-DAD) on a reversed-phase C18 column (Eclipse Plus C18, 150 × 4.6 mm, 5 µm), maintained at 30 °C. The mobile phase consisted of water (solvent A) and acetonitrile (solvent B), both containing 0.1% formic acid. Elution was performed using a gradient, starting at 15% B, increasing to 35% B at 20 min and to 50% B at 25 min, followed by re-equilibration. The flow rate was set at 1.0 mL/min and the injection volume was 10 µL.

Diode-array detection was performed at 280 nm for quantitative analysis, while full UV–visible spectra were acquired in the range 190–600 nm to support identification. Flavanone glycosides were identified by comparison of retention times and UV–visible spectral characteristics with those of authentic HPLC-grade reference standards analyzed under the same chromatographic conditions. Quantification was performed using external standard calibration curves.

The same analytical approach was subsequently applied to biological samples after appropriate sample preparation and, where required, enzymatic hydrolysis, as detailed in Section *Discussion*. Full method validation, including linearity, sensitivity, precision, and accuracy in the investigated matrices, is reported in Section *Method validation*.

#### Accelerated stability study and shelf-life estimation

An accelerated stability study was conducted on the capsule formulation in accordance with the ICH Q1A (R2) guideline (International Council for Harmonisation of Technical Requirements for Pharmaceuticals for Human Use, 2003). Samples were stored under accelerated conditions (40 °C/75% relative humidity) for 6 months and analyzed at predefined time points (0, 2, 4, and 6 months).

At each time point, capsules were opened, and the content was accurately weighed and subjected to solvent extraction before chromatographic analysis. Briefly, the capsule content was dissolved in dimethyl sulfoxide (DMSO), vortex-mixed to ensure complete dispersion, and subsequently diluted with a methanol/water mixture (60:40, *v*/*v*) acidified with formic acid (0.1%). The suspension was sonicated for 30 min, vortex-mixed, and then centrifuged. The supernatant was collected and appropriately diluted before HPLC-DAD analysis.

The content of the main flavanone markers (eriocitrin, neoeriocitrin, naringin, and neohesperidin) was quantified at each time point using the validated HPLC-DAD method described in Section *Analytical characterization of flavanone glycosides*. Results were expressed as percentage degradation relative to the initial content (time zero).

Under accelerated conditions, the four markers showed highly similar degradation profiles. For clarity, a single representative degradation profile based on mean values was used for kinetic analysis. Hesperidin, which exhibited a markedly lower degradation, was evaluated separately and excluded from the averaged profile.

Degradation kinetics were described using an apparent zero-order model, allowing estimation of the degradation rate constant (k, expressed as %·month^-1^) by linear regression constrained through the origin. A degradation threshold of 10% was selected as the end-of-life criterion, and the corresponding time under accelerated conditions (t_40_) was calculated.

Shelf-life at ambient temperature (25 °C) was estimated using a Q10-based extrapolation model, assuming a Q10 value of 2 and a 15 °C temperature difference. Real-time stability studies at ambient temperature are ongoing to confirm the estimated shelf-life.

### Human study

#### Subjects

Six healthy Caucasian volunteers (4 males and 2 females), aged 25 and 60 years, with a body mass index (BMI) of 18.5–24.9 kg/m^2^, were enrolled at the Section of Food, Clinical Nutrition and Drug Sciences, University of Rome “Tor Vergata”, between June and July 2025. All participants were non-smokers, did not consume alcohol regularly, and were in good health, with no history of cancer, cardiovascular, or other chronic systemic diseases.

Exclusion criteria were acute or chronic inflammatory, metabolic, or gastrointestinal disorders; infectious or autoimmune diseases; pregnancy; use of hormonal therapies (including oral contraceptives or hormone replacement therapy); use of medications or nutraceuticals affecting inflammatory or metabolic status.

All volunteers reported stable dietary habits and body weight for at least three months before enrollment. To minimize dietary interference, participants were instructed to abstain from fresh or processed *Citrus* products (including juices, preserves, and supplements) for two weeks before and throughout the study period.

#### Study protocol and ethical approval

The study was designed as an exploratory, single-arm intervention. Following eligibility screening, participants underwent a washout period and were instructed to abstain from dietary supplements, nutraceuticals, and *Citrus*-based foods to minimize interference with the investigated flavanone compounds.

After an overnight fast (10 h), each participant received a single oral dose (one FlavonAid™ capsule). Baseline assessments were performed prior to dosing to confirm eligibility and health status.

The protocol was approved by the Ethics Committee of the Calabria Region Central Area (Protocol No. 97, 20 April 2023) and conducted in accordance with the Declaration of Helsinki. All participants provided written informed consent.

#### Biological sample collection

Biological samples were collected according to a predefined schedule to characterize flavanone exposure and metabolic profiles after administration.

Fasting blood samples were collected at baseline (T0, pre-dose) and at 30, 60, 90, 180, and 240 min post-dose. Blood was collected in EDTA-coated tubes and centrifuged immediately. Plasma was separated, aliquoted, and stored at −80 °C until analysis.

The 4 h plasma sampling window was selected as an exploratory early post-dose interval to assess the initial systemic appearance of flavanone-related compounds, rather than to derive a complete pharmacokinetic profile. This choice reflects the known behavior of *Citrus* flavanones, which typically exhibit delayed plasma peaks due to intestinal and microbial processing prior to absorption.

Urinary excretion was assessed using cumulative samples collected over the intervals 0–8 h and 8–24 h post-dose, with a baseline (T0) sample. Urine was collected in containers holding sodium metabisulfite, aliquoted immediately after collection, and stored at −80 °C for metabolite analysis.

Fecal samples were collected at baseline (T0) and 24 h after capsule administration. Participants were provided with collection containers and instructed on standardized collection procedures. Upon delivery to the laboratory, fecal samples were immediately processed and stored at −80 °C until analysis.

### Bioanalytical methods

#### Sample preparation and enzymatic hydrolysis

Biological samples (plasma, urine, and feces) were subjected to enzymatic hydrolysis before extraction and chromatographic analysis. Syringic acid was added to all samples as an internal standard to monitor extraction efficiency and analytical variability. For plasma and urine samples, 100 µL aliquots were transferred into reaction tubes and mixed with 400 µL of sodium acetate buffer (0.1 M, pH 5.0); enzymatic hydrolysis was initiated by adding 10 µL of a β-glucuronidase/sulfatase solution from *Helix pomatia*, corresponding to a final activity of 2,000–5,000 U/mL, followed by incubation at 37 °C for 12–16 h. Fecal samples were processed after cryogenic grinding. Briefly, 100 mg of powdered feces were mixed with 1 mL of sodium acetate buffer (0.1 M, pH 5.0) and incubated with 20 µL of β-glucuronidase/sulfatase solution, corresponding to a final enzymatic activity of 5,000–10,000 U/mL, at 37 °C for 16 h. After hydrolysis, plasma and urine samples were subjected to protein precipitation by adding 250 µL of an acetonitrile/methanol mixture (1:1, v/v), vortex-mixed for 3 min and centrifuged at 12,000 rpm for 10 min at 4 °C; the supernatant was transferred to a new 1.5 mL Eppendorf tube, filtered through a 0.22 µm nylon filter, and evaporated to dryness under a nitrogen stream at room temperature (RT). The residue was reconstituted with 100 µL of acetonitrile/water (1:1, *v*/*v*), vortex-mixed for 1 min and centrifuged again (12,000 rpm, 10 min, 4 °C). The clarified extract was transferred to an autosampler vial and 10 µL injected into the HPLC–DAD system. Hydrolyzed fecal samples were extracted by adding 250 µL of an acetonitrile/methanol mixture (1:1, *v*/*v*), vortex-mixed for 3 min, and centrifuged at 12,000 rpm for 10 min at 4 °C; the supernatant was transferred to a new 2 mL Eppendorf tube and evaporated under nitrogen at RT. The residue was reconstituted with 200 µL of acetonitrile/water (1:1, *v*/*v*), vortex-mixed for 1 min and, when necessary, sonicated and maintained at 40 °C for 4 min to ensure complete dissolution, then filtered through 0.22 µm nylon filters, transferred to autosampler vials with inserts, and 10 µL were injected into the HPLC–DAD system.

#### Quantification approach and reporting as glycoside equivalents

After hydrolysis, aglycone forms (eriodictyol for eriocitrin and neoeriocitrin, naringenin for naringin, and hesperetin for hesperidin and neohesperidin) were quantified using authentic aglycone standards (purity ≥98, Extrasynthese, Genay, France). Quantified aglycones include both (i) aglycones present in the matrix and (ii) aglycones released from phase II conjugates after *in vitro* hydrolysis.

Accordingly, parent compounds detected in plasma represent the sum of intact flavanone glycosides, directly quantified using reference standards (purity ≥98, Extrasynthese, Genay, France), and a fraction calculated from quantified aglycones.

Quantified aglycones reflect the total aglycone pool, including both free aglycones and those released from conjugated forms. For dose comparison, plasma results are expressed as glycoside equivalents (ng/mL).

For dose comparison and partial dose-recovery assessment in urine and feces, aglycone amounts were back-calculated and expressed as equivalents of the administered parent flavanone glycosides (mg-eq), assuming a 1:1 M ratio between each glycoside and its corresponding aglycone. Conversion was performed according to the following equation:

$$mg-eq\;\left(glycoside\right)=\frac{\mu mol\;aglycone\;x\;MW\;glycoside}{1000}$$

Accordingly, “parent compounds” in urinary and fecal results represent glycoside equivalents derived from flavanone glycosides, include both directly quantified glycosides and fractions derived from free and conjugated aglycones.

In contrast, total shared metabolites (phenolic acids) were quantified using reference standards (purity ≥98, Extrasynthese, Genay, France) and subsequently summed, as individual phenolic acids cannot be unequivocally attributed to a specific parent flavanone, being common downstream metabolites of multiple investigated flavanones.

#### HPLC-DAD and LC-DAD-ESI-MS/MS analyses

Flavanone in biological samples were quantified by high-performance liquid chromatography coupled with diode-array detection (HPLC–DAD), under the chromatographic conditions described above. The method was applied across all investigated matrices.

Additionally, liquid chromatography coupled with electrospray ionization tandem mass spectrometry (LC–ESI–MS/MS) was used to support compound identification in complex samples. UV–visible spectra were acquired in the range 190–600 nm, and chromatograms were recorded at selected wavelengths (260, 292, 330, and 370 nm) to monitor phenolic compounds with different absorption features.

Mass spectrometric analyses were performed using an ion trap mass spectrometer equipped with an ESI source operated in positive and negative ion modes. Full-scan spectra were acquired over the m/z range 90–1000, and collision-induced dissociation (CID) MS/MS experiments were performed to obtain diagnostic fragmentation patterns.

Compound identification was achieved by combining evaluation of retention times, UV–visible absorption features, and mass spectral data. For compounds with available reference standards, identification was confirmed by direct comparison. Additional constituents were tentatively identified based on literature data and comparison with spectral databases, including SpectraBase®, PhytoHub, ReSpect, MassBank, and PubChem. Instrument performance was verified by replicate injections, with retention time variability within acceptable limits (≤2%).

#### Method validation

The HPLC–DAD method used for the quantitative determination of flavanone glycosides and related metabolites in biological samples was validated according to its intended analytical application.

Linearity was evaluated by external calibration using authentic HPLC-grade standards, including administered flavanone glycosides and measured metabolites. Calibration curves were constructed over appropriate concentration ranges. Linearity was confirmed by satisfactory correlation coefficients and consistent responses.

Method sensitivity was assessed by determining limits of detection and quantification according to the ICH guideline Q2 (R1) (International Council for Harmonisation of Technical Requirements for Pharmaceuticals for Human Use, 2003) approach, based on the standard deviation of the response and the slope of the calibration curve. Limits of detection (LOD) and limits of quantification (LOQ) values were estimated accordingly and may fall below the lowest concentration included in the validated linearity range. The concentration interval of 10–1,000 ng/mL represents the validated working range used for linearity assessment and selection of quality control levels. Quality control levels were selected to represent low, intermediate, and high concentrations within this range, to assess method performance across the working range in each matrix.

Precision was evaluated as intra and inter-day variability by repeated analysis of quality control samples at different levels and expressed as relative standard deviation (RSD). Accuracy was assessed by recovery experiments using spiked blank matrices.

Matrix effects were evaluated by comparing solvent–based and matrix-matched calibration curves. No significant endogenous interference was observed at analyte retention times.

Method robustness was assessed by evaluating minor variations in chromatographic conditions on retention times and peak responses. Overall, the method proved suitable for reliable quantitative determination of the targeted flavanone glycosides and metabolites in plasma, urine, and fecal samples.

### Statistical analysis

Given the exploratory nature of the study and the small sample size (*n* = 6), quantitative data are reported as median and interquartile range [Q1–Q3, IQR]. Regression analyses for the accelerated stability study, including zero-order kinetic modeling and estimation of the degradation rate constant (k_40_), were performed using SigmaPlot (Systat Software Inc., San Jose, CA, USA).

Principal component analysis (PCA) was performed on individual urinary and fecal recovery data (Ae 0–24 h, µmol). Prior to analysis, data were log10 (x + 1) transformed using, mean-centered, and autoscaled (unit variance) to account for differences in magnitude. PCA was performed using JMP® Pro 14 (SAS Institute Inc., Cary, NC, USA), and principal components were interpreted based on explained variance and loadings.

## Results

### Validation and analytical performance of the method

**FIGURE 1:**
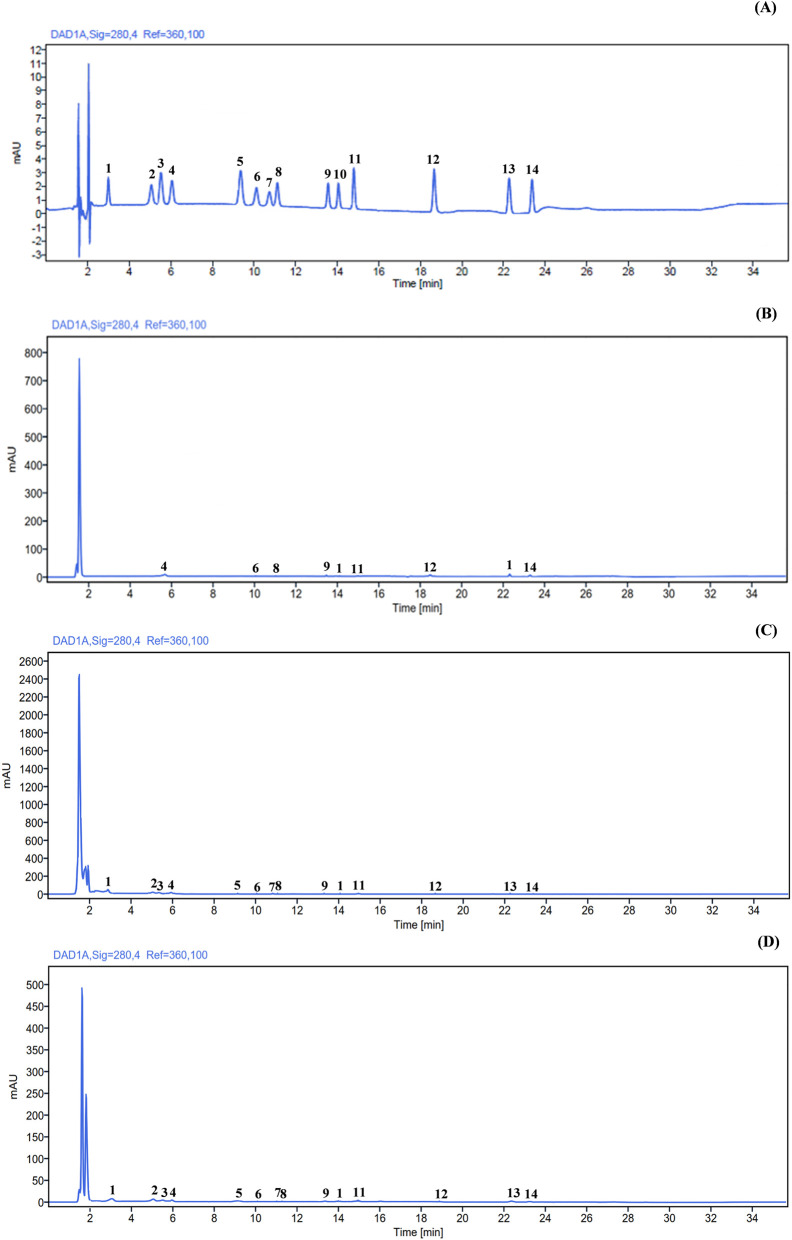
Representative chromatograms of a mixed standard solution (1 μg/mL) containing all identified compounds, including the internal standard (syringic acid) **(A)**, and of the three matrices: plasma **(B)**, urine **(C)**, and feces **(D)**. Analyte peaks are labeled according to their chromatographic elution order: (1) protocatechuic acid, (2) caffeic acid, (3) vanillic acid, (4) syringic acid, (5) *p*-coumaric acid, (6) eriocitrin, (7) ferulic acid, (8) neoeriocitrin, (9) naringin, (10) hesperidin, (11) neohesperidin, (12) eriodictyol, (13) naringenin, and (14) hesperetin.

The HPLC–DAD method for the quantitative determination of flavanone glycosides and related metabolites was successfully validated in urine, plasma, and fecal matrices (Figure 1). Validation results are summarized in Table 1.

**TABLE 1:** Validation parameters of the HPLC-DAD method for determination of flavanone glycosides and related metabolites in plasma, urine, and fecal samples.

Analyte	RT	Linearity range (ng/mL)	R^2^	LOD (ng/mL)	LOQ (ng/mL)	Intra-day precision (RSD %)	Inter-day precision (RSD %)	Recovery (%)
Urine								
Protocatecuic acid	3.00	10–1,000	0.999	1.05	3.19	0.33	5.15	99.59
Caffeic acid	5.31	10–1,000	1.000	0.09	0.28	2.87	8.89	98.19
Vanillic acid	5.44	10–1,000	1.000	0.11	0.32	4.42	9.17	97.73
*p*-Coumaric acid	9.36	10–1,000	1.000	0.43	1.29	1.43	4.48	99.30
Eriocitrin	10.12	10–1,000	0.999	1.69	5.12	3.81	8.09	97.39
Ferulic acid	10.94	10–1,000	1.000	0.12	0.35	4.17	7.06	97.59
Neoeriocitrin	11.13	10–1,000	0.999	0.92	2.78	4.36	7.76	98.40
Naringin	13.56	10–1,000	0.999	1.38	4.19	3.19	6.81	98.44
Hesperidin	14.06	10–1,000	0.999	1.82	5.53	4.85	6.27	97.71
Neohesperidin	14.80	10–1,000	0.999	1.62	4.91	3.82	7.20	98.00
Eridictiol	18.66	10–1,000	0.999	1.26	3.82	3.07	8.89	98.54
Naringenin	22.27	10–1,000	0.999	0.89	2.71	2.20	6.85	97.07
Hesperetin	23.37	10–1,000	0.999	1.17	3.54	2.87	9.26	98.93
Plasma								
Protocatecuic acid	3.01	10–1,000	0.999	1.58	4.78	0.55	8.76	95.19
Caffeic acid	5.32	10–1,000	0.999	0.13	0.40	4.59	14.22	94.39
Vanillic acid	5.44	10–1,000	0.999	0.14	0.42	5.30	11.00	94.33
*p*-Coumaric acid	9.37	10–1,000	0.999	0.68	2.06	2.15	6.72	96.29
Eriocitrin	10.13	10–1,000	0.999	2.03	6.14	4.95	10.52	95.92
Ferulic acid	10.94	10–1,000	0.999	0.20	0.60	5.00	8.47	94.88
Neoeriocitrin	11.13	10–1,000	0.999	1.38	4.17	5.23	9.31	95.77
Naringin	13.57	10–1,000	0.999	2.07	6.28	3.83	8.17	94.17
Hesperidin	14.08	10–1,000	0.999	2.55	7.74	5.82	7.52	94.79
Neohesperidin	14.81	10–1,000	0.999	2.10	6.38	4.97	9.36	95.04
Eridictiol	18.67	10–1,000	0.999	1.89	5.73	4.30	12.45	94.37
Naringenin	22.27	10–1,000	0.999	1.43	4.33	3.52	10.96	94.66
Hesperetin	23.37	10–1,000	0.999	1.98	6.01	4.88	15.75	94.98
Feces								
Protocatecuic acid	3.02	10–1,000	0.998	3.94	11.95	0.82	12.88	91.89
Caffeic acid	5.32	10–1,000	0.999	0.39	1.19	7.17	22.23	89.59
Vanillic acid	5.44	10–1,000	0.997	0.37	1.13	11.05	22.91	87.33
*p*-Coumaric acid	9.37	10–1,000	0.999	1.50	4.54	3.58	11.20	90.23
Eriocitrin	10.13	10–1,000	0.999	4.66	14.13	9.52	20.23	87.25
Ferulic acid	10.95	10–1,000	0.998	0.55	1.68	10.42	17.65	88.89
Neoeriocitrin	11.13	10–1,000	0.999	3.03	9.17	10.90	19.40	88.47
Naringin	13.57	10–1,000	0.998	4.77	14.44	7.99	17.02	89.17
Hesperidin	14.07	10–1,000	0.997	5.62	17.02	12.12	15.67	87.74
Neohesperidin	14.81	10–1,000	0.999	4.63	14.03	9.55	18.01	88.37
Eridictiol	18.67	10–1,000	0.997	3.97	12.03	7.68	22.22	88.7
Naringenin	22.27	10–1,000	0.998	3.0	9.10	5.50	17.13	87.67
Hesperetin	23.38	10–1,000	0.999	4.36	13.22	7.18	23.16	88.93

Excellent linearity was observed for all analytes across the concentration range (10–1,000 ng/mL) in all matrices, with determination coefficients (R^2^) consistently ≥ 0.997. Linearity was confirmed for both administered flavanone glycosides and measured metabolites, supporting its applicability to multi-analyte determination in complex matrices.

Sensitivity was highest in urine, with LOD ranging from 0.09 to 1.82 ng/mL and LOQ from 0.28 to 5.53 ng/mL. LOD and LOQ values were higher in plasma, reflecting greater matrix complexity, with LOD ranging from 0.13 to 2.55 ng/mL and LOQ from 0.40 to 7.74 ng/mL. The highest detection limits were observed in feces, where LOD and LOQ values ranged from 0.37 to 5.62 ng/mL and from 1.13 to 17.02 ng/mL, respectively. Despite this increase, sensitivity remained adequate for reliable quantification in all matrices.

Precision (intra- and inter-day RSD%) was satisfactory for all analytes. In urine, intra-day precision was excellent, with RSD values generally below 5%, with inter-day values below 10%. In plasma, intra-day RSD values were <6% and inter-day values <12%, with slightly higher variability observed for a limited number of analytes. In feces, intra-day precision remained below 12% for all compounds, while inter-day RSD values reached approximately 23%, consistent with the higher heterogeneity and complexity of this matrix.

Accuracy (recovery) was satisfactory across all matrices. Mean recovery values ranged from 97% to 100% in urine, from 94% to 96% in plasma, and from 87% to 92% in fecal samples.

The decrease in recovery from urine to plasma and feces reflects increasing matrix complexity without compromising analytical performance. Overall, the validation proved sensitive, precise, and accurate for the simultaneous determination of flavanone glycosides and related metabolites in urine, plasma, and feces, and suitable for application in human intervention studies involving complex matrices.

### Stability and shelf-life

Under accelerated storage conditions (40 °C/75% RH), the formulation exhibited limited degradation. The four monitored flavonoid markers (eriocitrin, neoeriocitrin, naringin, and neohesperidin) showed similar degradation profiles throughout the six-month study period. Therefore, a representative mean profile was used for clarity, while hesperidin was evaluated separately due to its lower degradation.

**FIGURE 2:**
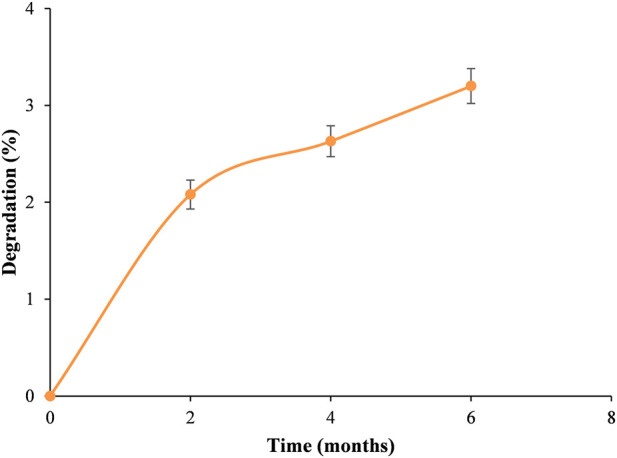
Representative accelerated stability profile of the main flavanone markers of the capsule formulation stored at 40 °C/75% RH for 6 months. Eriocitrin, neoeriocitrin, naringin, and neohesperidin showed similar degradation profiles; therefore, a mean degradation profile is shown. Error bars indicate variability. Hesperidin showed minimal degradation and was evaluated separately.

Mean cumulative degradation was 2.08% at 2 months, 2.63% at 4 months, and 3.20% at 6 months (Figure 2).

The degradation trend was approximately linear, supporting an apparent zero-order kinetic model.

Linear regression of the mean dprofile yielded a degradation rate constant at 40 °C (k_40_) of 0.605% per month^-1^. Using a 10% degradation threshold as end–of–life criterion, the estimated time to reach the degradation threshold under accelerated conditions was 16.5 months.

Shelf-life at ambient temperature (25 °C) was estimated using a Q10-based extrapolation model, assuming a Q10 value of 2. This resulted in an estimated shelf-life of approximately 47 months (∼3.9 years). Hesperidin showed minimal degradation and was considered at least as stable as the other flavonoids.

Overall, the stability profile supports the suitability of the formulation for long-term storage under standard conditions, pending confirmation by ongoing real-time studies.

### Plasma analysis

Flavanones in plasma were quantified using the validated HPLC–DAD method (Section *Analytical characterization of flavanone glycosides*). Analyte-specific LOD and LOQ values established during validation were applied. For consistency across biological matrices, plasma concentrations were expressed as glycoside equivalents (nmol/L). Although baseline (time 0) samples were collected, these data are not reported in Table 2, which is intentionally focused on post-dose measurements. Results were classified as not detected (ND) for concentrations below the LOD, detected but not quantifiable (DNQ) for concentrations between the LOD and LOQ, and quantifiable (Q) for concentrations equal to or above the LOQ. Given the expected low systemic exposure of flavanone glycosides, plasma data were used to describe detectability and concentration ranges over time and were not employed to derive classical pharmacokinetic parameters.

**TABLE 2:** Plasma detectability and concentration ranges of flavanones and flavanone-derived compounds in plasma, expressed as glycoside equivalents (nmol/L).

Flavanone	Time (h)	N	Min (nmol/L)	Max (nmol/L)	Median (nmol/L)	n ≥ LOD	n ≥ LOQ
Eriocitrin	0.5	6	9.17	112.01	37.53	4	3
	1.0	6	10.93	68.34	22.95	4	3
	1.5	6	5.21	41.00	7.86	5	1
	3.0	6	6.09	100.18	18.46	5	4
	4.0	6	19.95	100.18	58.25	3	3
Neoeriocitrin	0.5	6	10.07	171.81	113.29	5	4
	1.0	6	7.61	165.58	102.41	6	5
	1.5	6	44.98	149.01	107.96	4	4
	3.0	6	68.30	87.86	85.81	4	4
	4.0	6	83.13	107.96	103.87	4	4
Naringin	0.5	6	69.61	132.15	100.89	2	2
	1.0	6	56.15	160.92	119.25	4	4
	1.5	6	23.32	182.49	67.27	5	4
	3.0	6	14.37	163.66	37.21	6	3
	4.0	6	49.97	243.11	59.86	6	6
Neohesperidin	0.5	6	17.94	295.71	171.75	6	5
	1.0	6	23.85	255.72	166.46	6	6
	1.5	6	48.24	304.93	173.99	6	6
	3.0	6	34.92	305.45	150.92	6	6
	4.0	6	26.91	295.99	169.05	6	6
Hesperidin	0.5	6	9.96	457.66	119.97	4	2
	1.0	6	135.57	424.56	309.54	3	3
	1.5	6	21.01	483.86	239.98	4	3
	3.0	6	9.70	430.65	168.14	5	4
	4.0	6	33.79	369.92	211.95	4	4

Following single-dose administration, all five flavanones, expressed as glycoside equivalents, were detected in plasma samples collected between 0.5 and 4 h post-dose, at low concentrations with variable detectability. Overall, plasma exposure showed intermittent detection and limited quantifiability, with a substantial proportion of values falling within the DNQ range.

Across all post-dose samples (*n* = 30), detectability above LOD ranged from 20/30 to 30/30 samples depending on the analyte, whereas quantifiable concentrations (≥LOQ) were less consistently observed. Specifically, eriocitrin was detected above the LOD in 21/30 samples and quantified in 14/30; neoeriocitrin was ≥LOD in 23/30 samples and ≥LOQ in 21/30; naringin was detected in 23/30 samples and quantified in 19/30; neohesperidin showed the highest detectability, being ≥ LOD in all post-dose samples (30/30) and ≥LOQ in 29/30; hesperidin was ≥LOD in 20/30 samples and ≥LOQ in 16/30.

Plasma concentrations were generally close to analytical limits, particularly for eriocitrin, neoeriocitrin, and naringin, resulting in sporadic quantification and absence of clearly defined concentration–time profiles. Neohesperidin displayed the most consistent quantification across time points, whereas the other flavanones showed marked interindividual variability and intermittency. No evidence of compound accumulation was observed within the 4 h post-dose sampling window.

In plasma, phase I ring-fission metabolites (e.g., phenolic acids and related low-molecular-weight catabolites) were either detected at trace levels or found to be below the LOQ in most samples. Due to limited quantitative data, these compounds are not reported in the plasma results table, which therefore primarily reflects circulating parent-related compounds.

**FIGURE 3:**
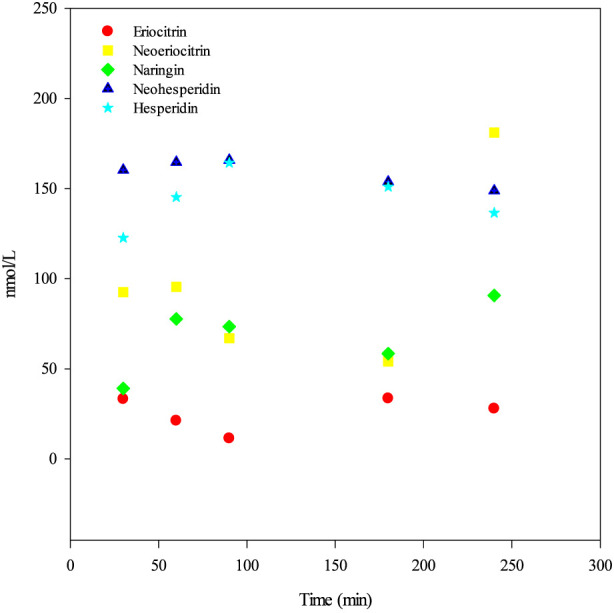
Dot plot of flavanone plasma concentrations over time after single-dose administration. Concentrations are expressed as glycoside equivalents (nmol/L). Each dot represents one individual subject (*n* = 6). The figure illustrates temporal distribution and interindividual variability.

Timepoint-specific concentration ranges, median values, and detectability metrics for each flavanone are reported in Table 2. A dot plot of individual plasma concentrations over time is provided in Figure 3 to facilitate visualization of temporal distribution and interindividual variability.

### Urinary excretion profile

Urinary concentrations of parent flavanones and metabolites were initially expressed as µg/mL. Concentrations were converted to molar units (µmol/L) using molecular weights as follows:

$$C_{\left(µmol/L\right)}=\frac{\left(C_{µg/mL}\right)}{MW}x1000$$

The total amount excreted in each interval (Ae, µmol) was calculated as:

$$Ae_{\mu mol}=C_{\mu mol/L}\times V_{L}$$

Urinary excretion is expressed as total amount over 24 h and reported as median with interquartile range [IQR, Q1–Q3] (*n* = 6). For each subject, total 24 h excretion was calculated by summing the amounts recovered in the two consecutive collection intervals.

Urinary analysis showed flavanone-related compounds for all glycosides over 24 h post-dose. After hydrolysis, both parent compounds and shared metabolites were detected.

Total urinary recovery reached a median of 335.30 µmol of flavanone-related compounds with marked interindividual variability (Table 3).

**TABLE 3:** Urinary amounts of flavanones and related compounds recovered over 24 h, with parent markers expressed as glycoside equivalents (µmol).

Compound	Ae urine 0–24 h (µmol) median	Q1–Q3 (µmol)
Hesperidin	0.07	0.05–0.09
Eriocitrin	2.84	0.76–74.59
Neoeriocitrin	0.28	0.25–0.30
Naringin	76.89	38.91–199.52
Neohesperidin	3.98	3.17–4.75
Total parent glycoside equivalents	170.65	103.34–266.88
Total shared metabolites	164.65	97.70–254.09
Total flavanone-related compounds (parent + metabolites)	335.30	201.04–520.97

Among the parent compounds, naringin showed the highest median recovery, whereas hesperidin showed the lowest recovery. Comparable contributions of parent compounds and shared metabolites were observed, with median values of 170.65 µmol and 164.65 µmol, respectively (Table 3). These results indicate that 24 h urinary excretion includes substantial amounts of both parent-derived and metabolite-derived compounds.

### Fecal analysis

Fecal concentrations of parent flavanones and metabolites were initially expressed as µg/g wet feces. Concentrations were converted to molar units using molecular weights, and excretion was expressed as total amount recovered over 24 h (Ae, µmol). Fecal data are reported as individual parent flavanones, total shared metabolites, and total flavanone-related compounds, without assigning metabolites to specific parent precursors.

Fecal analysis showed flavanone-derived compounds in all subjects over 24 h post-dose. After hydrolysis, fecal profiles showed both parent flavanones and shared metabolites. Quantitative assessment showed that metabolites accounted for the major fraction of total flavanone-related compounds.

Descriptive statistics for fecal amounts (µmol, total 24 h recovery), are reported in Table 4. Total parent compounds, total shared metabolites, and total flavanone-related compounds were calculated at the individual level and summarized. Among the parent flavanones, neohesperidin showed the highest median recovery, whereas naringin showed the lowest recovery. Overall, shared metabolites contributed more than parent compounds to the fecal flavanone-related pool.

**TABLE 4:** Fecal amounts of flavanones and related compounds recovered over 24 h, with parent markers expressed as glycoside equivalents (µmol), reported as median [IQR, Q1–Q3] (*n* = 6).

Compound	Ae feces 0–24 h (µmol) median	Q1–Q3 (µmol)
Hesperidin	1.39	1.22–3.02
Eriocitrin	1.43	0.94–2.24
Neoeriocitrin	1.08	0.13–1.47
Naringin	0.63	0.51–3.00
Neohesperidin	6.24	5.37–10.02
Total parent glycoside equivalents	10.49	9.52–15.97
Total metabolites (shared)	29.49	24.62–35.01
Total flavanone-related compounds (parent + metabolites)	35.87	35.11–50.99

### Principal component analysis

**FIGURE 4:**
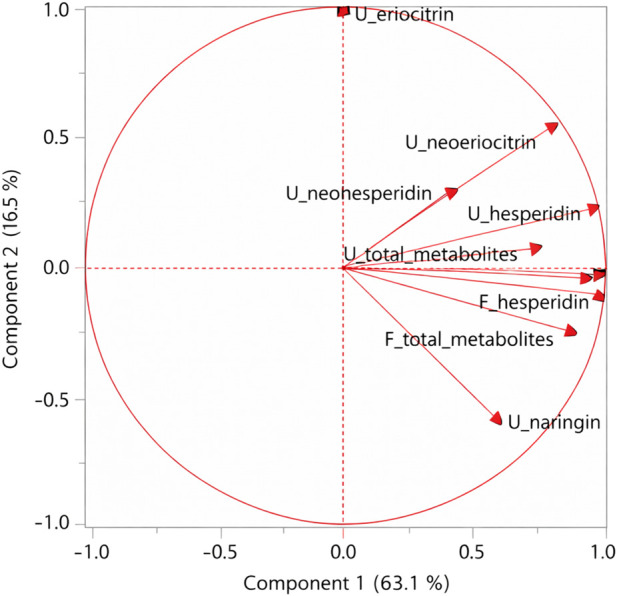
PCA correlation circle of flavanone-related compounds. Loading plot showing urinary (U) and fecal (F) parent glycoside equivalents and total shared metabolites recovered over 0–24 h. PCA was performed on individual recovery data (Ae, µmol) after log10 (x + 1) transformation, mean-centering, and autoscaling (unit variance) using JMP® Pro 14. The first two principal components explained 79.6% of the total variance (PC1: 63.1%; PC2: 16.5%). Urinary and fecal variables are labeled as U and F, respectively.

To explore multivariate relationships among urinary and fecal outputs, PCA was performed on parent glycoside equivalents and total metabolite recovery (Figure 4).

The first two principal components explained 79.6% of the total variance (PC1: 63.1%; PC2: 16.5%), indicating that the bidimensional model adequately described the dataset.

PC1 accounted for most of the variance and showed positive loadings for most variables, including urinary and fecal flavanones and total metabolites. PC1 mainly reflected overall recovered flavanone-related material rather than matrix-specific processes. The proximity of urinary and fecal counterparts of the same compound (e.g., hesperidin and naringin) suggests coordinated handling across compartments.

PC2, explaining an additional 16.5% of the variance, allowed partial compound-level differentiation. Urinary eriocitrin showed a strong positive loading, whereas urinary naringin showed a negative loading, indicating divergent contributions of specific flavanones to the overall excretion profile. This separation reflects compound-dependent differences in intestinal processing and metabolic transformation.

Overall, PCA results indicate that flavanone disposition is largely governed by intestinal metabolism, with coordinated urinary and fecal elimination reflecting a shared metabolic framework rather than independent matrix-specific behavior.

### Mass balance of administered flavanone glycosides

A mass balance assessment over 24 h was performed by comparing the administered dose (174 mg) with recovered amounts, expressed as glycoside equivalents and estimated from parent-related compounds quantified after hydrolysis.

Values are reported as median [IQR, Q1–Q3] and were calculated at the individual level using subjects with paired urine and feces collections (*n* = 6), followed by statistical summarization. Given the negligible plasma exposure, plasma data were not included in the mass balance calculation. Metabolite were quantified separately and were not incorporated into the dose-equivalent assessment.

Within 0–24 h, a fraction of the administered flavanone glycosides was recovered (Table 5).

**TABLE 5:** Mass balance of administered flavanone glycosides over 24 h, expressed as glycoside equivalents (µmol).

PC^*^	Urine	Feces	Total	Total
	(µmol)			(mg-eq)
HES	0.00 [0.00–0.03]	1.39 [1.22–3.02]	1.39 [1.25–3.02]	0.85
NHE	3.84 [2.94–4.13]	6.24 [5.37–10.02]	10.37 [8.31–15.92]	6.33
NAR	100.95 [52.83–232.38]	0.63 [0.51–3.00]	101.46 [55.84–268.38]	58.90
ERI	0.76 [0.09–2.84]	1.43 [0.94–2.24]	4.27 [2.24–75.53]	2.55
NER	0.00 [0.00–0.23]	1.08 [0.13–1.47]	1.08 [0.13–1.69]	0.65
Total	103.89 [103.16–237.40]	10.49 [9.52–15.97]	113.65 [113.41–292.67]	67.56 [66.16–170.55]

* PC, parent compounds, expressed as glycoside equivalents (µmol) estimated after enzymatic hydrolysis. Values represent total parent-related compounds, including intact glycosides and fractions derived from quantified aglycones (free and conjugated forms after hydrolysis). Parent compounds: hesperidin (HES), neohesperidin (NHE), naringin (NAR), eriocitrin (ERI), and neoeriocitrin (NER).

Overall, approximately 39% of the administered dose was recovered as hydrolyzed parent glycoside equivalents within 24 h (median value, *n* = 6). Within this fraction, urinary recovery exceeded fecal recovery, indicating renal elimination as the predominant early excretion route.

## Discussion

The present study provides an integrated evaluation of human exposure to *Citrus* flavanones using a multi-matrix approach combining plasma, urinary, and fecal analyses. This multi-matrix approach is particularly relevant for flavanone glycosides, whose biopharmaceutical behavior is characterized by limited absorption of the intact glycosylated forms and extensive intestinal and microbial processing (Manach et al., 2003; Manach et al., 2005; Brett et al., 2009). As a result, reliance on plasma concentration–time profiles alone may substantially underestimate overall exposure and fail to capture key aspects of *in vivo* disposition.

Plasma measurements in the present study indicated very limited systemic exposure to flavanone-related compounds expressed as glycoside equivalents. Although detectable concentrations were observed across the post-dose window, values were generally low and frequently close to the LOD and LOQ values. Detectability was intermittent and highly variable across subjects, and no reproducible concentration–time profiles consistent with classical pharmacokinetics were observed. These findings are fully consistent with previous human studies reporting low and variable plasma concentrations of flavanone glycosides following *Citrus* product consumption (Manach et al., 2003; Brett et al., 2009; Najmanová et al., 2020). These findings indicate that plasma represents a complementary matrix, providing qualitative rather than comprehensive information on exposure.

Among the compounds investigated, neohesperidin exhibited the highest frequency of quantifiable plasma concentrations. This observation may reflect compound-specific differences in glycosidic structure, gastrointestinal stability, and susceptibility to enzymatic and microbial processing, which could favor a slightly higher probability of systemic appearance compared with other flavanone glycosides (Brett et al., 2009; Murota et al., 2018). Overall, systemic exposure was limited and transient.

This interpretation should be considered in light of the 4 h sampling window, which may not capture delayed circulating profiles.

In contrast, urinary analysis provided more robust insight into the *in vivo* handling of flavanone glycosides. Measurable amounts of flavanone-related compounds were recovered in urine within 24 h following administration, indicating partial absorption and renal elimination. Both hydrolyzed parent flavanones and shared low-molecular-weight phenolic metabolites contributed substantially to urinary excretion, indicating combined effects of limited systemic appearance and extensive metabolism. These findings align with established models of flavanone metabolism, whereby intestinal deglycosylation precedes absorption and phase II metabolism, followed by efficient renal clearance of absorbed fractions (Manach et al., 2005; Williamson and Clifford, 2018).

Because several phenolic metabolites quantified in urine are shared among different flavanone precursors, their measurement provides a pragmatic, semi-quantitative description of flavanone-derived metabolic output without implying precursor specificity. In this context, urine emerges as a useful matrix for characterizing overall flavanone disposition, whereas plasma captures only a limited and transient fraction of systemic exposure. Integration of urinary data with fecal excretion profiles is therefore essential to obtain a comprehensive picture of flavanone handling in humans.

Fecal analysis further complements urinary findings and provides direct evidence of sustained intestinal processing of flavanone glycosides. Detectable amounts of flavanone-derived compounds were recovered in feces over the 24 h post-dose period, with shared metabolite-related species accounting for the predominant fraction of the fecal output. This pattern reflects limited absorption of intact glycosides and ongoing intestinal and microbial biotransformation, largely occurring in the colon (Selma et al., 2009; Murota et al., 2018). Because several fecal metabolites may originate from multiple flavanone precursors, results were conservatively reported without assigning individual metabolites to specific parent compounds, ensuring a robust and reproducible description of fecal excretion patterns.

The combined urinary and fecal data indicate a disposition profile characterized by measurable short-term urinary elimination together with substantial intestinal processing.

To further explore the integrated handling of flavanone-related compounds across biological compartments, multivariate analysis by PCA was applied to individual 0–24 h urinary and fecal recovery data. PCA provided an orthogonal view of the relationships among parent flavanone equivalents and shared metabolites, allowing evaluation of coordinated versus compound-specific patterns.

The first principal component mainly captured the magnitude of overall recovered material and explained most of the variance, with positive loadings for most urinary and fecal variables. This pattern indicates that interindividual variability was largely driven by differences in total flavanone-related recovery rather than by matrix-specific elimination routes, supporting integrated intestinal–systemic handling. The close spatial proximity of urinary and fecal counterparts of the same flavanones further suggests coordinated downstream processing across biological compartments.

The second principal component highlighted compound-level differentiation that could not be attributed solely to glycosidic linkage, as naringin, neohesperidin, and neoeriocitrin all share a neohesperidoside moiety. Instead, this separation likely reflects combined effects of sugar linkage and aglycone structure. Differences in B-ring hydroxylation and methoxylation patterns—such as catechol-type substitution in eriodictyol derivatives versus monohydroxyl or methoxylated structures in naringenin and hesperetin derivatives—are known to influence susceptibility to intestinal microbial transformation and downstream metabolic pathways (Selma et al., 2009; Murota et al., 2018; Williamson and Clifford, 2018). These structural features may therefore contribute to compound-dependent variations in intestinal processing efficiency and excretion patterns.

The close clustering of neoeriocitrin and neohesperidin in the PCA loading space supports a largely shared handling across matrices, consistent with common upstream intestinal processing steps such as deglycosylation. In contrast, the divergent orientation of naringin along PC2—despite its neohesperidoside structure—suggests that sugar linkage alone is insufficient to explain flavanone disposition and highlights the role of aglycone structure and microbiota-dependent transformation (Manach et al., 2005; Najmanová et al., 2020).

Overall, PCA reinforces the interpretation that flavanone disposition is governed by an integrated intestinal metabolic framework, with coordinated urinary and fecal elimination reflecting downstream handling of shared metabolic pathways rather than independent matrix-specific pharmacokinetic behavior.

Taken together, the coordinated urinary and fecal patterns highlighted by PCA provide a coherent framework for quantitative interpretation of dose recovery through mass balance analysis.

Mass balance analysis showed that a substantial fraction (∼39%) of the administered dose was recovered within 24 h as hydrolyzed parent flavanones in urine and feces, with approximately 39% of the dose accounted for as parent equivalents. This partial recovery does not indicate complete systemic absorption, but rather as evidence of continued intestinal metabolism and delayed elimination beyond the observation window. Such a profile reflects the dynamic interplay between intestinal deglycosylation, microbial-mediated biotransformation, and absorptive transport processes that govern flavanone disposition in humans.

Importantly, partial systemic recovery does not contradict a potential local mode of action. Even in the presence of measurable absorption and renal elimination, substantial luminal exposure may persist during gastrointestinal residence, supporting interactions with the intestinal epithelium and the gut microbiota. This kinetic behavior differs from that of rapidly and completely absorbed small molecules and supports a model in which flavanone glycosides exert combined local and limited systemic effects. In this context, the observed profile is particularly compatible with applications targeting intestinal disorders, where sustained luminal exposure together with controlled systemic availability may represent a desirable pharmacological balance (Selma et al., 2009; Williamson and Clifford, 2018).

Taken together, the present findings indicate that human exposure to *Citrus* flavanones cannot be adequately described by plasma data alone. The integration of plasma, urinary, and fecal measurements provides a more physiologically meaningful assessment of flavanone disposition, highlighting the central role of intestinal processing and delayed elimination. This multi-matrix framework supports further investigation of flavanone-based formulations as candidates for locally acting intestinal interventions and provides a rational basis for future studies employing extended collection periods and disease-oriented models to fully characterize long-term metabolic dynamics.

**FIGURE 5:**
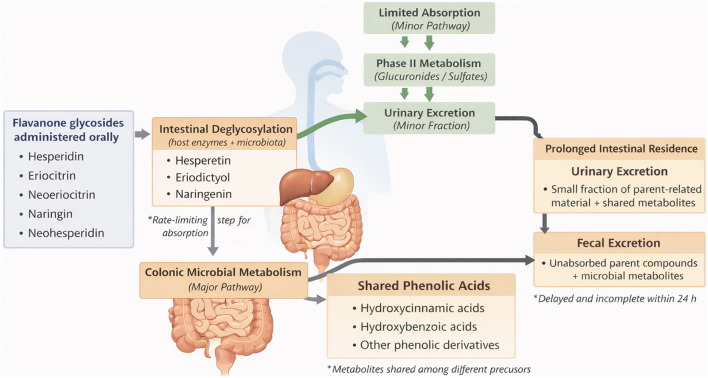
Schematic overview of the intestinal metabolism and excretion pathways of flavanone glycosides in humans, illustrating intestinal deglycosylation, microbial transformation, limited systemic absorption, and urinary and fecal elimination.

The integrated multi-matrix data support a model of flavanone disposition characterized by intestinal processing, limited systemic exposure, and prolonged elimination (Figure 5). This framework reconciles low plasma detectability with measurable urinary and fecal recovery, highlighting the central role of intestinal metabolism and microbiota-driven transformation.

In addition to disposition and excretion, the stability and dose reliability of the administered formulation supports the interpretation of human exposure data. The accelerated stability study demonstrated minimal degradation of the main flavanone glycosides over 6 months under stressed conditions, with an extrapolated shelf-life of approximately 4 years at RT. These findings indicate that the administered dose can be considered chemically stable and reproducible over time, minimizing variability. This aspect is particularly relevant in nutraceutical research, where insufficient characterization of product stability often complicates the interpretation of human intervention outcomes (Bajaj et al., 2012; Blessy et al., 2014). In the present study, the combination of stability data and analytical characterization supports the reliability of the declared flavanone dose and strengthens the link between administered formulation and observed biological profiles.

The observed excretion patterns further underscore the central role of the intestinal microbiota in determining flavanone fate in humans. Flavanone glycosides are poorly absorbed in their intact form and undergo extensive microbial-mediated biotransformation in the distal intestine, including deglycosylation, ring fission, and conversion into low-molecular-weight phenolic acids shared among multiple precursors (Selma et al., 2009; Murota et al., 2018; Williamson and Clifford, 2018). The predominance of shared metabolites in fecal samples and their substantial contribution to urinary output are consistent with this paradigm and highlight the microbiota as a key determinant of interindividual variability. Microbial variability likely contributes to interindividual differences. Although microbiota composition was not directly assessed in the present study, the data strongly support its role as a major modulator of flavanone bioavailability and metabolic fate.

From a translational perspective, the involvement of the intestinal microbiota reinforces the concept that flavanone-based nutraceuticals may exert relevant biological effects through local and microbiota-mediated mechanisms, rather than through sustained systemic exposure. This is particularly pertinent for conditions involving the gastrointestinal tract, where prolonged luminal residence and microbial transformation may contribute to epithelial signalling, immune modulation, and metabolic crosstalk. The kinetic profile observed here, characterized by limited plasma exposure, measurable urinary elimination, and persistent fecal recovery, aligns with this mechanistic framework.

Several limitations of the present study should be acknowledged. The relatively small sample size and exploratory design limit generalizability of the findings and do not allow the application of formal pharmacokinetic modeling. In addition, the 24 h collection window primarily captures early elimination processes and does not fully account for delayed microbial metabolism and prolonged excretion, which may extend beyond the observation period. Finally, the absence of direct microbiota profiling precludes correlation analyses between microbial composition and individual metabolic patterns.

These limitations are typical of early-phase nutraceutical studies and are counterbalanced by the depth of analytical characterization and the integrated multi-matrix approach employed. Importantly, extended sampling periods, direct assessment of gut microbiota composition and functionality, and application of this experimental framework in disease-specific populations are currently ongoing within parallel and follow-up investigations. These studies are expected to further elucidate the relative contributions of local versus systemic pathways under pathological conditions and to refine exposure–response relationships relevant for clinical efficacy.

Despite its exploratory design, the data establish a robust methodological and conceptual framework for investigating flavanone disposition in humans and highlight the importance of stability, intestinal metabolism, and multi-compartment analysis.

## Conclusion

This study provides an integrated assessment of human exposure to *Citrus* flavanone glycosides following administration of a novel nutraceutical formulation, combining plasma, urinary, and fecal analyses with analytical characterization and stability evaluation.

Systemic exposure to intact flavanone glycosides was limited and variable, whereas substantial intestinal processing and measurable urinary and fecal elimination were observed.

The multi-matrix approach highlights the limitations of plasma–only assessment and underscores the central role of intestinal metabolism and delayed elimination in determining flavanone exposure. Multivariate analysis supports this view, indicating that variability is primarily driven by total recovered flavanone-related material, with secondary contributions from compound-specific structural features and microbial processing. The ∼39% recovery within 24 h, together with persistent fecal detection and extensive formation of shared metabolites, supports a model of coordinated intestinal processing with limited systemic exposure rather than classical pharmacokinetics.

The demonstrated chemical stability of the formulation supports the reliability of dose–exposure relationships.

Overall, these findings establish a robust framework for investigating flavanone-based nutraceuticals in humans and support further studies targeting conditions where sustained intestinal exposure with limited systemic availability is desirable.

## Data Availability

The original contributions presented in the study are included in the article/supplementary material, further inquiries can be directed to the corresponding author.
